# Draft Genome Sequence of Plant Growth-Promoting Rhizobacterium *Pantoea* sp. Strain AS-PWVM4

**DOI:** 10.1128/genomeA.00947-13

**Published:** 2013-12-05

**Authors:** Indu Khatri, Sukhvir Kaur, Usha Devi, Navinder Kumar, Deepak Sharma, Srikrishna Subramanian, Adesh K. Saini

**Affiliations:** CSIR-Institute of Microbial Technology, Chandigarh, Indiaa; Shoolini University of Biotechnology and Management Sciences, Department of Biotechnology, Bajhol, Solan, HP, Indiab

## Abstract

Nonpathogenic *Pantoea* spp. have been shown to confer biofertilizer and biocontrol activities, indicating their potential for increasing crop yield. Herein, we provide the high-quality genome sequence of *Pantoea* sp. strain AS-PWVM4, a Gram-negative motile plant growth-promoting rhizobacterium isolated from a pomegranate plant. The 4.9-Mb genome contains genes related to plant growth promotion and the synthesis of siderophores.

## GENOME ANNOUNCEMENT

The rhizospheric region of the plant root contains beneficial microbes that improve plant growth directly through their biofertilizer and phytostimulation activities or indirectly via their biocontrol activities. *Pantoea* spp., which are nonpathogenic plant epiphytic bacteria, have been reported as useful bacteria originating from edible plants (1–4). *Pantoea agglomerans*, isolated as a symbiotic bacterium from wheat and rice, was reported to fix nitrogen and solubilize inorganic phosphorus (1), is involved in indole-3-acetic acid and siderophore production (2), and is capable of plant growth promotion (3, 4). Some *Pantoea* strains have been developed as biological control agents for plant pathogens (5–7). *P. agglomerans* strain CPA-2, isolated from apple and pear, was found to be effective against *Botrytis cinerea*, *Penicillium expansum*, and *Rhizopus stolonifer* (8). *Pantoea* sp. strain AS-PWVM4, isolated from the rhizosphere of *Punica granatum*, exhibits phosphate solubilization, ammonia production, and hydrogen cyanide (HCN) and siderophore production, and it confers activity against a *Fusarium* sp., a fungal plant pathogen of pomegranates (S. Kaur, U. Devi, I. Khatri, D. Sharma, S. Subramanian, and A. K. Saini, unpublished data).

The genome of *Pantoea* sp. AS-PWVM4 was sequenced using the Illumina-HiSeq 1000 technology. Sequencing resulted in 31,931,152 paired-end reads (insert size of 350 bp) 101 bp in length. A total of 31,678,261 high-quality reads with approximately 640× coverage were assembled with CLC bio wb6 (word size, 35; bubble size, 55) to obtain 48 contigs (N_50_, 218,003 bp). The genome-finishing module of CLC bio, followed by the SSPACE version 2.0 scaffolder (9) and GapFiller version 1-10 (10), was used. The gap-filled scaffolds thus obtained were broken at the gaps to obtain 46 contigs (N_50_, 213,320 bp) of 4,990,872 bp, with an average G+C content of 54%. Functional annotation was carried out by Rapid Annotations using Subsystems Technology (RAST) (11), tRNAs were predicted by tRNAscan-SE 1.23 (12), and rRNA genes were predicted by RNAmmer 1.2 (13). The genome contains 3 rRNA genes (5S-23S-16S) and 75 aminoacyl-tRNA synthetase genes. A total of 4,525 coding regions (2,248 genes transcribed from the positive strand and 2,277 genes from the negative strand) were found in the genome, of which 3,752 (83%) were functionally annotated. The genome coding density is 88%, with an average gene length of 942 bp. The annotated genome has 97 genes responsible for motility and chemotaxis, including 16 genes for flagellar motility. Fifty genes are responsible for phosphorus metabolism. Twenty-nine genes are osmotic stress-responsive genes, including 4 for osmoregulation and 69 for oxidative stress, for a total of 165 genes in this organism that are responsible for stress response.

A functional comparison of the genome sequences available on the RAST server revealed the closest neighbor of *Pantoea* sp. AS-PWVM4 to be *Pantoea* sp. strain At-9b (score, 542), followed by *Pantoea ananatis* LMG 20103 (score, 493), *Pantoea* sp. strain αB (score, 426), and *Pantoea vagans* C9-1 (score, 407).

### Nucleotide sequence accession numbers.

This whole-genome shotgun project has been deposited at DDBJ/EMBL/GenBank under the accession no. ASZC00000000. The version described in this paper is the first version, ASZC01000000.
